# *In Vivo* Selection of Resistant *E. coli* after Ingestion of Milk with Added Drug Residues

**DOI:** 10.1371/journal.pone.0115223

**Published:** 2014-12-15

**Authors:** Richard Van Vleck Pereira, Julie D. Siler, Rodrigo Carvalho Bicalho, Lorin D. Warnick

**Affiliations:** Department of Population Medicine and Diagnostic Sciences, College of Veterinary Medicine, Cornell University, Ithaca, NY, United States of America; University of South Florida College of Medicine, United States of America

## Abstract

Antimicrobial resistance represents a major global threat to modern medicine. *In vitro* studies have shown that very low concentrations of drugs, as frequently identified in the environment, and in foods and water for human and animal consumption, can select for resistant bacteria. However, limited information is currently available on the *in vivo* impact of ingested drug residues. The objective of our study was to evaluate the effect of feeding preweaned calves milk containing antimicrobial drug residues (below the minimum inhibitory concentration), similar to concentrations detected in milk commonly fed to dairy calves, on selection of resistant fecal *E. coli* in calves from birth to weaning. At birth, thirty calves were randomly assigned to a controlled feeding trial where: 15 calves were fed raw milk with no drug residues (NR), and 15 calves were fed raw milk with drug residues (DR) by adding ceftiofur, penicillin, ampicillin, and oxytetracycline at final concentrations in the milk of 0.1, 0.005, 0.01, and 0.3 µg/ml, respectively. Fecal samples were rectally collected from each calf once a week starting at birth prior to the first feeding in the trial (pre-treatment) until 6 weeks of age. A significantly greater proportion of *E. coli* resistant to ampicillin, cefoxitin, ceftiofur, streptomycin and tetracycline was observed in DR calves when compared to NR calves. Additionally, isolates from DR calves had a significant decrease in susceptibility to ceftriaxone and ceftiofur when compared to isolates from NR calves. A greater proportion of *E. coli* isolates from calves in the DR group were resistant to 3 or more antimicrobial drugs when compared to calves in the ND group. These findings highlight the role that low concentrations of antimicrobial drugs have on the evolution and selection of resistance to multiple antimicrobial drugs *in vivo*.

## Introduction

The rapid development of antimicrobial resistance in the past few decades is considered one of the greatest global threats to modern medicine [1], [2]. Acceleration in the rise of antimicrobial resistance has widely been blamed on misuse and overuse of drugs in humans and food-producing animals, emphasizing the need for the judicious use of antimicrobials [3], [4]. In the dairy industry diseases have a great impact, resulting in treatment expenses and production losses such as lower milk yield and withholding of milk due to presence of drug residues [5]. Most antimicrobial drugs used to treat cows result in the milk from these animals being withheld from sale (waste milk) because of the presence of drug residues above the tolerance concentration established by the U.S. Food and Drug Administration (FDA). A tolerance level is a concentration determined by the FDA at which residues of a substance present in a food will have no harmful effects on the human consumers of the food product [6]. To decrease production losses due to waste milk, 33% of dairy farms in the United States feed preweaned calves waste milk [7]. Feeding pasteurized waste milk instead of milk replacer to preweaned calves has been shown to result in an estimated saving of $0.69 per calf per day [8].

In a recent study conducted by our research group, waste milk fed to calves was collected from several dairy farms in central New York and screened for drug residues [9]. The three most common drug residues identified were the following β-lactams: ceftiofur, penicillin G, and ampicillin. Although these β-lactams drugs were observed at concentrations above the tolerance levels for human consumption of milk established by the FDA, their concentrations were still below the minimal inhibitory concentration established for *E. coli* by the Clinical Laboratory Standard Institute (CLSI) [10]. Minimum inhibitory concentrations (MICs) are defined as the lowest concentration of an antimicrobial that will inhibit the visible growth of a microorganism after overnight incubation. Because selection of resistant bacteria has traditionally been assumed to occur at concentrations between the MIC of the susceptible wild type population and that of the resistant bacteria, the potential impact of feeding waste milk to calves on drug resistance has not been evaluated extensively. However, recent studies have shown that selection of resistant bacteria can occur at low antimicrobial drug concentrations by selecting for resistant bacteria with compensatory mutations that counterbalance the decreased fitness cost caused by resistance [11], [12].

Studies have reported that antimicrobial drugs at concentrations below the minimal inhibitory concentration (sub-MIC) can stimulate mutagenesis and recombination, leading to bacterial adaptation to various stresses, including antimicrobial pressure [13], [14]. An increase in mutagenesis can also result in heterogeneous increases in MIC of the bacteria across a range of antimicrobials, irrespective of the drug target [15]–[17]. Exposure of bacteria to sub-MICs has also been shown to significantly increase the frequency of transfer on mobile genetic elements (MGE), which can result in increased dissemination of antimicrobial resistance genes between bacteria. In a recent study, *E. coli* carrying the multidrug resistance plasmid pB10 was exposed to concentrations of tetracycline as low as 0.01 µg/mL, what was shown to significantly increase the transfer rate of this plasmid to enteric bacteria present in activated sludge from a wastewater treatment plant [18]. Furthermore sub-MICs of antimicrobial drugs could potentially increase the spread of antibiotic resistance genes (ARG) in the environment, which are currently considered an emerging environmental contaminant in lakes, rivers, and at drinking water treatment plants, representing a health hazard for human and animal health [19].

The development of a commensal microbiota carrying resistance to antimicrobials represents an important reservoir for resistance genes (resistome) that could be transferred to pathogenic bacteria, leading to the emergence of clinically problematic strains [20]. Moreover, antimicrobial treatment failure in a disease caused by pathogenic bacteria susceptible to a drug used for treatment could occur as a consequence of a resistant commensal microbiota through an umbrella effect. This umbrella effect was shown in a study by Perlin et al. (2009) while examining if β-lactamase-producing *E. coli* could protect ampicillin-sensitive cohorts of other species, particularly species that can cause human disease [21]. They observed that β-lactamase-secreting *E. coli* allowed for survival of a large number of ampicillin-sensitive cohorts of *Salmonella enterica* serovar Typhimurium. The *Salmonella* survivors remained sensitive to ampicillin when re-plated onto solid medium and there was no evidence of gene transfer, suggesting a protective effect by β-lactamase-secreting *E. coli*.

Few studies have evaluated the impacts of ingesting sub-MICs of antimicrobial drugs in an *in vivo* model, and many questions remain about the effects on the enteric microbiota and selection of antimicrobial resistance [15]. The objective of our study was to evaluate the effect of feeding preweaned dairy calves raw milk with residual concentrations (sub-MICs) of ampicillin, ceftiofur, penicillin, and oxytetracycline from birth to weaning on the selection of resistant *E. coli* in the feces.

## Material and Methods

### Ethics statement

Fecal samples were collected from calves (*Bos taurus*) that were housed on Cornell University facilities. The research protocol was reviewed and approved by the Institutional Animal Care and Use Committee of Cornell University (Protocol number: 2012–0090).

### Study design and sample collection

Randomized controlled feeding trials were conducted at the College of Veterinary Medicine, Cornell University (Ithaca, NY, USA) from June 2013 to March 2014. Three feeding trials were completed with a total of 10 male calves in each trial, with 5 calves belonging to each treatment group. All thirty calves enrolled in the trials were purchased from a local dairy farm and enrolled in the study on their date of birth. Control calves (n = 15) were enrolled in the trials concurrently with test calves (n = 15). At least one author in the study was involved in all calf collection at the farm. Upon collection, a physical examination was performed and calves were weighed. Additionally, the assignment of calves to study groups was done at the farm by pairing calves born on the same day by weight and using a coin toss to randomly allocate a calf as either a test or control. Once a calf was assigned to a treatment group it received an identification tag, which was placed in the right ear. All calves were fed 2–4 liters of maternal colostrum from individual cows within the first 4 h of life. Colostrum fed to calves in both milk feeding treatments originated from the dairy farm where calves were collected. At this farm cows were under the same management. After feeding of colostrum, calves were transported from the source farm to Cornell University.

Calves were individually housed in concrete box stalls to prevent contact between calves. Blood samples were collected from each calf in the first 24–48 hours of life, and the serum total protein was measured to assess adequacy of passive transfer. Control calves were fed raw milk without the addition of antimicrobial drugs (NR), and test calves were fed raw milk with the addition of low concentrations of ceftiofur, penicillin, ampicillin and oxytetracycline (DR). All calves were bucket fed one gallon of raw whole milk twice a day from birth to 6 weeks of age. Feedings occurred once in the morning and once in the afternoon with approximately 12 hour intervals between feedings. A non-medicated pelleted calf starter (18% crude protein, 3% crude fat, 8% crude fiber; DuMOR Calf Starter, Tractor Supply Co., Brentwood, TN) was offered from day 7 until day 42 of life up to a maximum of 1 kg/day. To prevent cross-contamination between calves, each calf stall had dedicated equipment and supplies, and all study personnel used personal protective equipment when entering each calf stall, which was changed after exiting the calf stall.

Single-use gloves were used to collect rectal fecal samples from each calf once a week starting at birth, prior to the first feeding in the trial (pre-treatment), until 6 weeks of age. No calves required treatment with therapeutic antimicrobial drugs during the trial.

### Spiking milk with drug residues

Raw milk used to feed calves was collected daily from the Cornell University College of Veterinary Medicine Dairy Farm. Antimicrobial stock solutions used to spike milk were prepared one week prior to each calf trial. Stocks were prepared by diluting powdered drugs in distilled water to a concentration of 100 µg/mL for ampicillin sodium salt, 1,000 µg/mL for ceftiofur sodium, 50 µg/mL for penicillin G sodium, and 3,000 µg/mL for oxytetracycline hydrochloride. Individual sterile cryovials with 2.28 ml of each stock solution for each antimicrobial drug were stored at −80°C until used [22]–[24]. Individual antimicrobial stocks (instead of a cocktail of drugs) for each antimicrobial drug were prepared to avoid potential interactions between different antimicrobials before adding it to the milk.

Milk containing drug residues fed to DR calves was prepared twice a day 10 to 20 minutes prior to feeding. For each drug, a tube containing 2.28 ml of antimicrobial stock solution was thawed at room temperature and added to a batch of 22.8 liters of raw milk which was stirred for 1 minute at approximately 400 RPM prior to feeding to calves. The final concentration of each antimicrobial drug in the milk fed to DR calves was calculated to be: 0.01 µg/ml of ampicillin sodium, 0.1 µg/ml of ceftiofur sodium, 0.005 µg/ml of penicillin G sodium, and 0.3 µg/ml of oxytetracycline hydrochloride. Because limited information is currently available on the impacts of waste milk on selection of resistance, drugs were pooled in the milk (instead of tested individually) to simulate what is expected to be observed on dairy farms. Milk fed to NR calves was also stirred for 1 minute at approximately 400 RPM prior to feeding, but without the addition of any antimicrobial and by using clean dedicated equipment to avoid drug contamination from milk fed to DR calves. After each feeding, equipment was thoroughly cleaned separately by milk feeding treatment group using a dedicated brush and hot water, liquid soap, and sodium hypochlorite (The Clorox Co., Oakland, CA). The selection of drugs and the concentration added to the milk was based on an article published by our research group where we screened waste milk withheld for sale at dairy farms in central New York [9]. In that study the most prevalent drugs detected by LC-MS/MS were ceftiofur (mean ± SE concentration  = 0.151±0.042 µg/mL), penicillin G (mean ± SE concentration  = 0.008±0.001 µg/mL), and ampicillin (mean ± SE concentration  = 0.472±0.43 µg/mL). In addition, one sample had detectable concentrations of oxytetracycline (0.01 µg/mL). Because of the high frequency use of this drug in dairy cattle, it was also added to the milk fed to DR calves [25]. Moreover, the tolerance and safe levels for drug residues in raw milk used for human consumption determined by the Federal Department of Agriculture (FDA) were also used to determine at what concentration the selected antimicrobial drugs should be added to the milk.

Raw milk used in the trial was screened daily for drug residues prior to its use in the study. Two commercial tests were used: New SNAP Beta-lactam Test Kit (IDEXX Laboratories Inc., Westbrook, ME) which detects penicillin (LOD = 0.005 ppm), ampicillin (LOD = 0.01), amoxicillin (LOD = 0.01 ppm), cephapirin (LOD = 0.02 ppm), and ceftiofur (LOD = 0.1 ppm) residues in raw milk; and SNAP Tetracycline Test Kit (IDEXX Laboratories Inc., Westbrook, ME) which detects tetracycline (LOD = 0.05 ppm), oxytetracycline (LOD = 0.05 ppm), and chlortetracycline (LOD = 0.1 ppm) residues in raw milk. During the entire trial all milk samples from the dairy farm tested negative for drug residues.

Milk spiked with antimicrobial drugs fed to DR calves was screened weekly for drug residues using the New SNAP Beta-lactam Test Kit and the SNAP Tetracycline Test Kit. As expected, all spiked milk samples tested positive. There are no current commercial test kits for detecting drug residues in cow colostrum. Additionally the commercial kits available for milk are not appropriate for colostrum because the high fat percentage and viscosity of colostrum compared to milk can interfere in the results of the test. Therefore colostrum fed to calves was not screened for drug residues. However, colostrum fed to calves in both milk feeding treatments originated from one dairy farm where cows were under the same management.

### Bacterial isolation, culture, and antimicrobial susceptibility testing

Individual fecal samples were streaked onto MacConkey agar plates on the day of collection and incubated overnight at 37°C. Up to three *E. coli* colonies were selected and stored in Luria-Bertani broth containing 20% glycerol at −80°C, as previously described [26]. All fecal samples had three *E. coli* colonies, except for a few samples collected at week 0, which were meconium samples. All samples collected at week 0 had at least one *E. coli* isolate isolated from feces. A total of 609 *E. coli* isolates were cultured from rectal fecal samples and tested for antimicrobial susceptibility.

Antimicrobial susceptibility of *E. coli* isolates was tested against a modified National Antimicrobial Resistance Monitoring System (NARMS) panel of 12 antimicrobial drugs. The susceptibility testing was done using a Kirby–Bauer disk diffusion agar assay in accordance with the guidelines published by the Clinical and Laboratory Standards Institute (CLSI) and methodology previously described [26]–[28]. Internal quality control was performed by inclusion of *E. coli* ATCC 25922, which was previously determined to be pansusceptible, and a previously characterized in-house *E coli* isolate known to have a *bla*_CMY-2_ gene and to be resistant to 9 of the antimicrobials tested. Antimicrobial susceptibility for all isolates was assessed using the following panel: ampicillin 10 µg, cefoxitin 30 µg, ceftiofur 30 µg, ceftriaxone 30 µg, chloramphenicol 30 µg, ciprofloxacin 5 µg, gentamicin 10 µg, nalidixic acid 30 µg, neomycin 30 µg, streptomycin 10 µg, tetracycline 30 µg and trimethoprim-sulfamethoxazole 23.75/1.25 µg. Results of the disk diffusion test for the internal quality control strains were within the anticipated standards. Susceptibility of the isolates to antimicrobial drugs was categorized as susceptible, intermediate, or resistant (SIR) by measuring the inhibition zone diameter according to interpretive criteria and breakpoints established by the CLSI guidelines [10].

### Statistical analyses

Descriptive analysis for the SIR distribution of *E. coli* isolates by antimicrobial drug for each feeding treatment group was done using PROC FREQ in SAS (SAS Institute Inc., Cary, NC). Descriptive analysis of *E. coli* resistance phenotypes and the proportion of isolates pansusceptible or resistant to 3 or more antimicrobial drugs for each weekly sampling was also done using PROC FREQ in SAS. In this study, phenotypic multidrug resistance was defined as isolates having resistance to ≥3 antimicrobial agents. Descriptive analysis for the comparisons of the average weight gain during the 6 weeks of the calf trial between NR calves and DR calves was conducted in SAS using PROC GLIMMIX.

To evaluate the effects of calf milk feeding treatment over time in weeks on the proportion of resistant *E. coli* per calf for each of the 12 antimicrobials tested, multivariable mixed logistic regression models were fitted to the data using the GLIMMIX procedure of SAS. The independent variables milk feeding treatment, time in weeks of sampling, and interactions were included in all models. The effect of animal identification nested within trial number was controlled in the models as a random effect. Adjusted probabilities (for all variables and interactions offered to each model) for antimicrobial resistance were obtained using the LSMEANS statement. This statistical model was also used to evaluate the effect of milk feeding treatment over time in weeks on the proportions of multidrug resistant isolates, where the only difference was that the dependent variable was the binary variable for classification of *E. coli* as resistant to 3 or more antimicrobial drugs or not.

For antimicrobial drugs with more than 10% of *E. coli* isolates within each treatment group classified as intermediate according to CLSI breakpoints, an additional analysis using the GLIMMIX procedure of SAS was conducted. In this model, the dependent variable was a categorical variable that classified isolates as susceptible or nonsusceptible (intermediate or resistant). This binomial variable was used in the analysis for two main reasons: to reduce erroneous analysis of the data caused by a high number of isolates classified as intermediate and where the CLSI breakpoints may not correlate well to acquisition of resistant genes, and to focus the analysis on the presence or absence of isolates that are fully susceptible to the antimicrobial drugs tested. The independent variables milk feeding treatment, time in weeks of sampling, and interactions were included in all models. The effect of animal identification nested within trial number was controlled in the models as a random effect. Adjusted probabilities (for all variables and interactions offered to each model) for antimicrobial resistance were obtained using the LSMEANS statement.

To determine if there was a statistical difference between the diameter of the inhibition zones of *E. coli* at the calf level for each treatment group, generalized linear models were fitted to the data using the GLM procedure of SAS. For each antimicrobial drug, the calf level mean zone diameter value was calculated for each week using Proc Means in SAS. For the linear regression analysis, the independent variables milk feeding treatment, time in weeks of sampling, and interactions were included in all models. The effect of trial number was controlled in the models as a random effect. Adjusted means (for all variables and interactions offered to each model) for inhibition zone diameter were obtained using the LSMEANS statement.

## Results

### Descriptive data

The mean birth weight for the NR calves was 45.3 kg (range: 31–53 kg) and for the DR calves the mean was 44 kg (range: 34–53 kg). Mean serum total protein (g/dL) for blood samples collected 24–48 hrs after birth was 5.5 (range: 5.0–6.2 g/dL) for the DR calves and 5.4 (range: 5.1–6.0 g/dL) for the NR calves. No calf in the study had failure of passive transfer (serum total protein <5 g/dL). Average weight gain during the 6 weeks of the calf trials was 1.1lbs (95% C.I. 0.9–1.4) for NR calves and 1.35lbs (95% C.I. 1.1–1.6) for DR calves. There was no significant difference in the average weight gain during the 6 weeks of the calf trials between NR calves and DR calves (*P*-value  = 0.3).

### Antimicrobial Resistance in *E. coli* isolates

**Figure 1:**
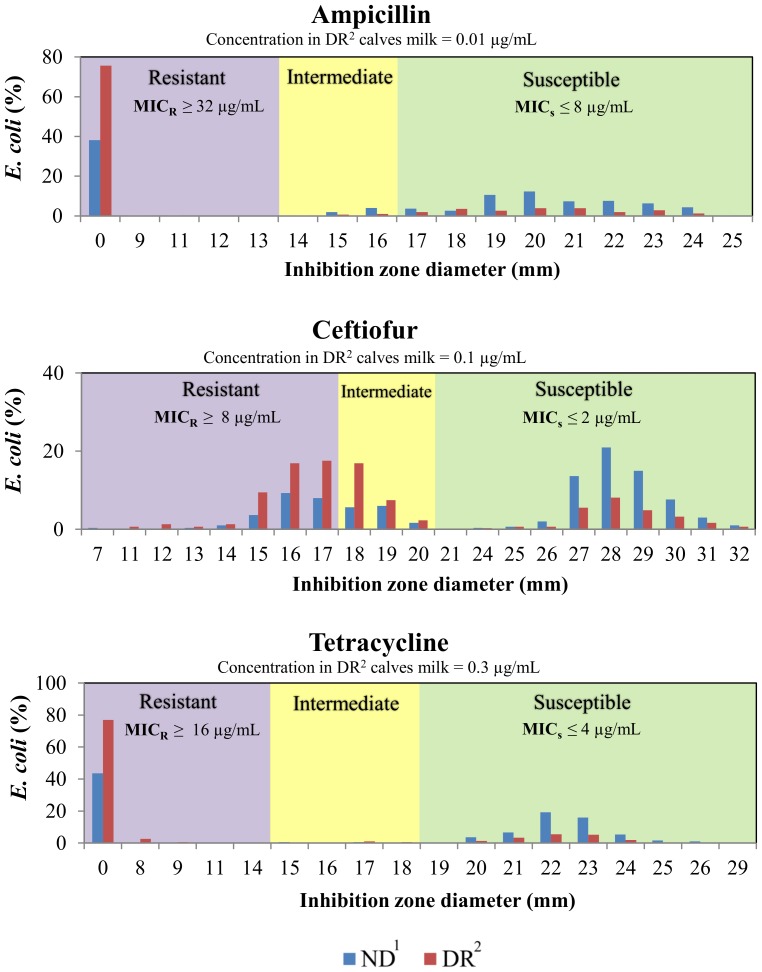
Zone diameter histogram of all *E. coli* cultured from fecal samples and tested for antimicrobial susceptibility (n = 609) to drugs added to the milk fed to DR calves. Data is stratified by milk feeding treatment group. Labeled shaded areas on graphs correspond to the zone diameter SIR classification according to Clinical and Laboratory Standards Institute (CLSI) breakpoints. The CLSI minimum inhibitory concentration breakpoints for *E. coli* classified as susceptible (MIC_s_) and resistant (MIC_R_) are displayed in graphs. 1. Calves fed raw milk without the addition of sub-MICs of antimicrobial drugs. 2. Calves fed raw milk with the addition of sub-MICs of ceftiofur, penicillin, ampicillin, and oxytetracycline from birth to 6 weeks of age.

**Figure 2:**
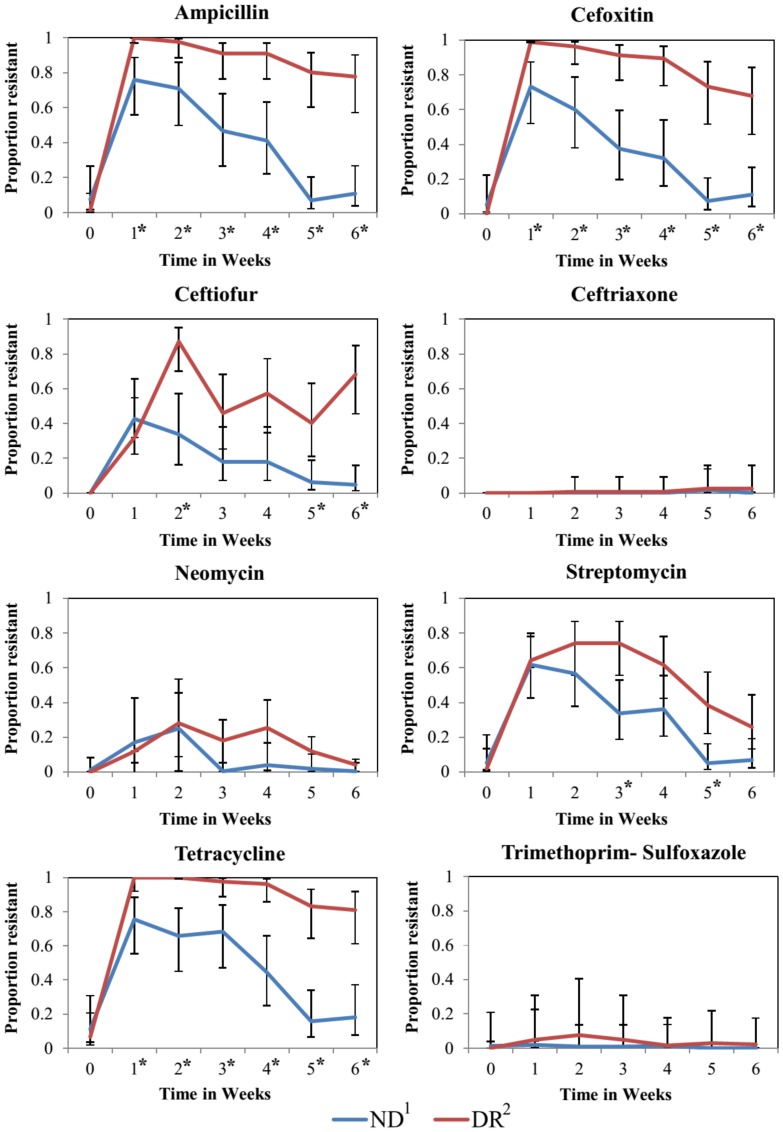
Proportion of resistant *E. coli* by milk feeding treatment at the calf level over time in weeks. Error bars represent 95% confidence interval of the least square mean. 1. Calves fed raw milk without the addition of sub-MICs of antimicrobial drugs. 2. Calves fed raw milk with the addition of sub-MICs of ceftiofur, penicillin, ampicillin, and oxytetracycline from birth to 6 weeks of age. *Sampling weeks where the proportion of resistance was significantly different between NR and DR.

**Figure 3:**
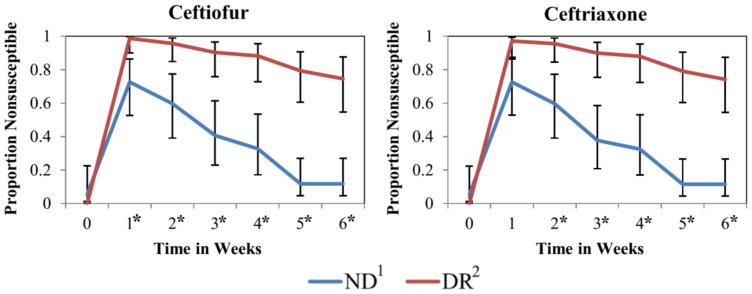
Proportion of *E. coli* nonsusceptible to ceftiofur and ceftriaxone by milk feeding treatment at the calf level over time in weeks. Error bars represent 95% confidence interval of the least square mean. 1. Calves fed raw milk without the addition of sub-MICs of antimicrobial drugs. 2. Calves fed raw milk with the addition of sub-MICs of ceftiofur, penicillin, ampicillin, and oxytetracycline from birth to 6 weeks of age. *Sampling weeks where the proportion of resistance was significantly different between NR and DR.

SIR distribution of *E. coli* and mean disk diffusion zone diameter by milk feeding treatment group are shown on table 1. Additionally, fig. 1 has a histogram for the zone diameter of all *E. coli* cultured from fecal samples and tested for antimicrobial susceptibility to three of the four drugs added to the milk of DR calves (ampicillin, ceftiofur and tetracycline). No difference was observed in the proportion of nonsusceptible isolates of NR and DR for the pre-treatment sample (week 0) for any of the antimicrobial drugs tested. For ampicillin (weeks 1 to 6), cefoxitin (weeks 1 to 6), ceftiofur (weeks 2, 5 and 6), streptomycin (weeks 3 and 5) and tetracycline (weeks 1 to 6), there was a significantly greater proportion of resistant isolates from DR calves when compared to NR calves (Fig. 2). Additionally for ceftiofur and ceftriaxone, which had more than 10% of isolated *E. coli* within each treatment group classified as intermediate, we observed a significantly greater proportion of non-susceptible isolates from DR calves when compared to NR calves (Fig. 3).

**Table 1:** SIR distribution of *E. coli* and mean disk diffusion zone diameter (ZD) by milk feeding treatment group.

Antimicrobials	NR^1^				DR^2^			
	S,%	I,%	R,%	Mean ZD	S,%	I,%	R,%	Mean ZD
**Ampicillin**	55	6	39	12	22	2	76	4
**Cefoxitin**	65	0	35	20	28	0	72	13
**Ceftiofur**	64	13	23	24	25	27	48	19
**Ceftriaxone**	64	35	1	27	26	71	3	21
**Ciprofloxacin**	99	0	1	36	99	0	1	35
**Chloramphenicol**	88	0	12	21	87	1	12	21
**Gentamicin**	99	0	1	24	100	0	0	24
**Nalidixic Acid**	99	0	1	25	100	0	0	25
**Neomycin**	87	0	13	19	77	2	21	19
**Streptomycin**	61	8	31	14	47	4	49	11
**Tetracycline**	54	2	44	12	17	2	81	4
**TMS** *	89	1	10	24	84	0	15	23

1. Percent distribution by antimicrobial of *E. coli* from calves fed raw milk without the addition of sub-MICs of antimicrobial drugs.

2. Percent distribution by antimicrobial of *E. coli* from calves fed raw milk with the addition of sub-MICs of ceftiofur, penicillin, ampicillin, and oxytetracycline from birth to 6 weeks of age.

* Trimethoprim-sulfamethoxazole.

**Figure 4:**
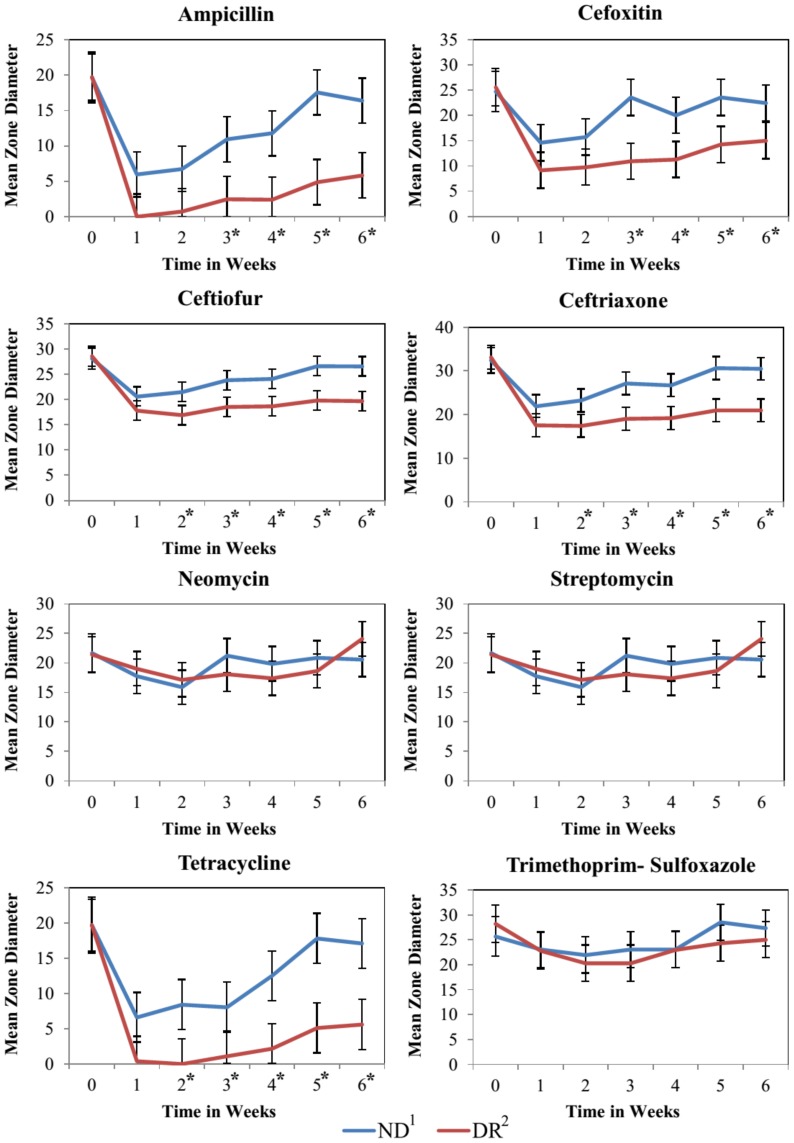
The effect of milk feeding treatment on the least square means of the zone diameter of *E. coli* at the calf level over time in weeks. Error bars represent 95% confidence interval of the least square mean. 1. Calves fed raw milk without the addition of sub-MICs of antimicrobial drugs. 2. Calves fed raw milk with the addition of sub-MICs of ceftiofur, penicillin, ampicillin, and oxytetracycline from birth to 6 weeks of age. *Sampling weeks where the mean zone diameter was significantly different between NR and DR.

For both treatment groups, calves in the study had a peak in the proportion of resistant *E. coli* at one to two weeks of age for the majority of the antimicrobial drugs tested (Fig. 2). The inhibition zone diameter for ampicillin, cefoxitin, ceftiofur, ceftriaxone, and tetracycline was significantly wider for these drugs in isolates from DR calves when compared to NR calves (Fig. 4).

### Distribution of multidrug resistant *E. coli* and resistance phenotypes

Of the 270 *E. coli* isolates obtained from DR fecal samples collected during weeks 1 to 6, 6% were pansusceptible (isolates non-resistant to all antimicrobial drugs tested) and 84% were resistant to three or more antimicrobials. The most common resistance phenotype from DR calves was ampicillin-cefoxitin-ceftiofur-tetracycline, which was present in 17% of isolates (Table 2). Of the 270 *E. coli* isolates from NR fecal samples collected during weeks 1 to 6, 46% were pansusceptible and 37% were resistant to three or more antimicrobials. The most common resistance phenotype from NR calves was ampicillin-cefoxitin-ceftiofur-neomycin-streptomycin-tetracycline, which was present in 10.3% of isolates (Table 2).

**Table 2:** Ranking of the most common antimicrobial resistant phenotypes (ARP) among 540 *E. coli* from fecal samples collected from week 1 to week 6.

Drug Resistant phenotypes	DR Rank^1^	NR Rank^2^	DR ^1^ %, n	NR^2^ %, n
AMP-FOX-TIO-TET	1	6	17 (47)	2 (6)
AMP-FOX-TIO-NEO-STR-TET	2	1	12 (32)	10 (28)
AMP-FOX-TET	3	10	11 (29)	1 (4)
AMP-FOX-TIO-CHL-STR-TET-COT	4	3	9 (25)	8 (21)
TET	7	2	5 (13)	9 (24)
** PANSUSCEPTIBLE**			6 (15)	46 (27)

AMP, Ampicillin; FOX, cefoxitin; TIO, ceftiofur; CHL, chloramphenicol; GEN, gentamicin; NEO, neomycin; STR, streptomycin; COT, trimethoprim sulfamethoxazole; TET, tetracycline.

1. Ranking or percent of ARP for isolates from calves fed raw milk with the addition of sub-MICs of ceftiofur, penicillin, ampicillin, and oxytetracycline from birth to 6 weeks of age.

2. Ranking or percent of DRP for isolates from calves fed raw milk without addition of sub-MICs of antimicrobial drugs.

**Figure 5:**
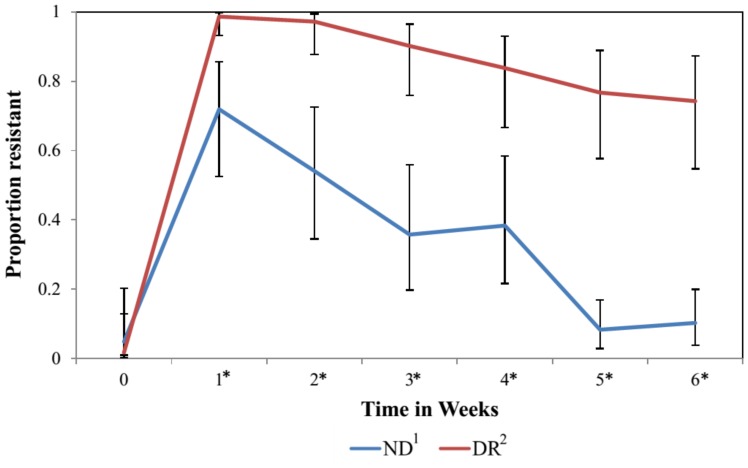
Proportion of *E. coli* resistant to 3 or more antimicrobial drugs by milk feeding treatment overtime in weeks. Error bars represent 95% confidence interval of the least square mean. 1. Calves fed raw milk without the addition of sub-MICs of antimicrobial drugs. 2. Calves fed raw milk with the addition of sub-MICs of ceftiofur, penicillin, ampicillin, and oxytetracycline from birth to 6 weeks of age. *Sampling weeks where the the proportion of resistant isolates resistance to 3 or more antimicrobial drugs was significantly different between NR and DR.

From week 1 to week 6, a significantly greater proportion of isolates from DR calves was resistant to 3 or more antimicrobial drugs compared to isolates from NR calves (Fig. 5). No significant difference was observed in the proportion of isolates resistant to 3 or more antimicrobial drug between NR calves and DR calves for week 0 (the pre-treatment samples).

## Discussion

The presence of low concentrations of antimicrobials and of antimicrobial resistance genes in natural environments such as rivers and soils, and in foods and water for human and animal consumption have been widely reported [9], . Our understanding of the impacts of low concentrations of drugs on antimicrobial resistance is still limited, although *in vitro* studies have indicated that selection and maintenance of resistant bacteria can occur [32]. The present study observed that calves fed milk with added antimicrobial drugs at very low concentrations had a significantly higher proportion of isolates resistant to three β-lactams drugs, an aminoglycoside drug, and a tetracycline drug when compared to calves fed milk without the addition of drugs (Fig. 2). The traditional concept of antimicrobial resistance has been suggested to be the selection of resistant bacteria when exposed to drugs at concentrations above the minimum inhibitory concentration (MIC) of the susceptible bacteria [33]. Above the MIC the susceptible strain will be out-competed by strains with phenotypic resistance to that drug. According to the CLSI, the MIC breakpoint for resistant bacteria in the *Enterobacteriaceae* family (which includes *E. coli*) for tetracycline, ceftiofur, and ampicillin is 16 µg/mL, 8 µg/mL, and 32 µg/mL, respectively (Fig. 1). The final concentration of tetracycline, ceftiofur, and ampicillin in the milk fed to DR calves was 0.3 µg/mL, 0.1 µg/mL, and 0.01 µg/mL, respectively. Therefore the concentration of tetracycline, ceftiofur, and ampicillin in the milk fed to DR calves was respectively 53, 80, and 3200 times below the MIC breakpoint for resistant *E. coli* [34]. Our findings challenged the conventional concentration window for selection of resistance and indicate that exposure of commensal *E. coli* to sub-MIC of antimicrobials drugs in preweaned dairy calves can cause selection of resistant bacteria.

In a study by Gullberg et al. (2011), isogenic pairs of susceptible and tetracycline resistant (*Tn*10*dTet*) *S. enterica* serovar Typhimurium were grown in the presence of tetracycline at concentrations many times below the MIC [32]. Their findings were similar to ours, observing that tetracycline concentrations up to 60 times below the MIC for the susceptible bacteria reduced the growth rate in the susceptible strain without any apparent effect on the resistant strain. These findings suggest that for most antimicrobial drugs, susceptible bacteria will experience a reduction in growth even when exposed to concentrations many times below the MIC. This sub-MIC selection window is commonly called the minimum selective concentration (MSC), and is defined as the lowest concentration of an antimicrobial drug that still selects for a given resistance determinant [35]. The MSC is directly associated with the fitness cost of the resistance determinant conferring competitive advantage to the resistant strain in relation to the susceptible strains. This means that if the fitness cost of resistance is too elevated, its presence will not necessarily result in a lower MSC for the resistant bacteria. For example, in the same study by Gullberg et al. (2011), antimicrobial sensitivity tests were conducted for ciprofloxacin using a pair of isogenic *E. coli* with either the mutation *gyA*(D87N) or *gyA*(S83L) [32]. Although both mutations select for resistance to ciprofloxacin, the MSC for *E. coli* with *gyA*(D87N) and *gyA*(S83L) was 0.0025 ug/ml and 0.00001 ug/ml, respectively. The cause of is difference is that the fitness cost for the mutation *gyA*(D87N) is 3%, while for *gyA*(S83L) it is 0.2%. These findings reinforce the argument that the effect of resistance determinants on fitness is a major factor affecting the MSC [35]. Moreover, this raises concerns about the exposure of a microbiota to antimicrobial drugs at sub-MIC, as it could cause selection of a sub-population of strains carrying resistance with low fitness cost that could contribute to the persistence of these bacteria in the commensal microbiota, even after exposure to antimicrobial drugs had ceased.

More than 35 percent of isolates in both feeding treatment groups were classified as intermediate for the third-generation cephalosporin ceftriaxone (Table 1). The intermediate zone is considered a buffer zone that should prevent small, uncontrolled, technical factors from causing major discrepancies in interpretation, and typically only a limited proportion of isolates are expected to be allocated in this category. Classification of many isolates as intermediate has also been reported by other researchers who suggested that species-specific breakpoints may be needed for these drugs to allow accurate prediction of the MIC equivalent of the zone diameters [36]. Although DR calves did not have a significantly higher proportion of isolates resistant to ceftriaxone when compared to ND calves (Fig. 2), they did have a significantly higher proportion of isolates nonsusceptible (isolates classified as intermediate or resistant) to ceftriaxone when compared to isolates from ND calves (Fig. 3). A lack of a species-specific clinical breakpoint for ceftriaxone, resulting in a high number of isolates classified as intermediate, could have been the reason for this discrepancy. The decrease in susceptibility to ceftriaxone could also be noted by a significantly narrower zone diameter for this drug in isolates from DR calves when compared to ND calves (Fig. 4). In our study, we compared the average zone diameter (ZD) of isolates at the calf level for each antimicrobial to allow detection of changes in susceptibility within a range that would not lead to changes in SIR classification. In this analysis, significantly larger zone diameters for an antimicrobial drug did not necessarily mean that one group had more clinically resistant isolates, but instead showed a reduced susceptibility to that drug. Resistance to third-generation cephalosporins is mainly conferred by the AmpC-like CMY β-lactamases and by the CTX-M β-lactamases [37], [38]. The selection of a sub-population of *E. coli* isolates less susceptible to third generation cephalosporins from feeding calves milk with sub-MICs is of concern to human health as drugs in this class have been labeled as critically important to human medicine by the World Health Organization (WHO) [39].

A higher proportion of resistant isolates from DR calves for three β-lactams was not unexpected, since three out of four of the drugs added to the milk of DR calves belonged to this drug class. This also applies to tetracycline resistance, because oxytetracycline was added to the milk with drug residues. Mechanism of resistance to β-lactams and tetracycline is commonly due to plasmid-mediated acquisition of resistance genes [40]–[43]. In addition to the selection pressure for resistant bacteria, sub-MIC concentrations of antimicrobials can increase rates of homologous recombination and horizontal gene transfer of resistant genes [44]–[46]. For example, exposure of *Staphylococcus aureus* to sub-MICs of β-lactams was shown to stimulate a 1000-fold increase in the transfer of a plasmid conveying tetracycline resistance [47]. Moreover sub-MICs of antimicrobials in DR calves could have increased resistance in isolates from DR calves by favoring the transfer rate of plasmids between commensal *E. coli*, amplifying the spread of resistance to β-lactams and tetracyclines between microbes in the gut.

Although no aminoglycoside was added to their milk, DR calves had a higher proportion of *E. coli* resistant to streptomycin at weeks 3 and 5 when compared to NR calves (Fig. 2). Target modification by mutation of the 16S rRNA or ribosomal proteins can cause resistance to aminoglycosides [48], [49]. Additionally, mutation in genes controlling efflux pumps for aminoglycoside drugs can result in overexpression, reducing susceptibility to aminoglycosides [50]. Similarly, resistance to β-lactams can also occur by mutations that increase the chromosomal expression level of β-lactamase (*bla*) genes, which can extend the substrate specificity to important 3^rd^ and 4^th^ generation cephalosporins [51]. Sub-MICs of antimicrobial drugs have been shown to increase mutagenesis, which has been correlated with an increase in reactive oxygen species and induction of the SOS response in bacteria [13], [17]. A recent study by Gutierrez et al. (2013) that exposed *E. coli* to sub-MICs of ampicillin elucidated some of the mechanisms that result in increased mutagenesis [52]. This study showed that sub-MICs of ampicillin induced mutagenesis by the combined activities of both the error-prone DNA polymerase and the normal replicative DNA polymerase in the absence of an adequate mismatch repair system. The mismatch repair system is a DNA-repair system present in most organisms that recognizes and repairs erroneous DNA replication and recombination, and DNA damage [53]. Moreover the higher proportion of isolates resistant to streptomycin in DR calves could have been caused by higher mutagenesis induced by sub-MICs of antimicrobial drugs. Alternatively, selection of resistance to streptomycin could have been caused by a higher occurrence of a class 1 integron in *E. coli* from DR calves. Integrons are mobile DNA elements capable of encoding multiple resistance genes at once [54]. Characterization of class 1 integron-mediated antimicrobial resistance among *E. coli* from calves has revealed its capability to integrate multiple antimicrobial resistance genes including β-lactams and aminoglycosides [55]. Sub-MICs of antimicrobials could result in the selection of *E. coli* with class 1 integrons in the DR group, causing co-selection of resistance to streptomycin.

DR calves had a significantly greater proportion of *E. coli* resistant to 3 or more antimicrobial drugs when compared to NR calves (Fig. 5). Multidrug resistance caused by sub-MICs of antimicrobials has been shown to be conferred through many of the mechanisms already discussed such as increase in horizontal gene transfer (e.g. mediated through plasmids), mutagenesis, co-selection through mobilization of integrons with cassettes carrying antimicrobial resistant genes, and activation and overexpression of bacteria efflux systems [47], . Additionally, an *in vitro* study by Kohanski et al. (2010) observed that exposure of *E. coli* to sub-MICs of bactericidal antimicrobials could lead to multidrug resistance via radical-induced mutagenesis [15]. Moreover, a study by Braoudaki et al. (2004) observed a high degree of cross-resistance to a range of antimicrobials and biocides in *Salmonella* serovar Virchow and *E. coli* O157 when strains were repeatedly exposed to sub-MICs of antimicrobials agents [57]. Although their study could not determine the specific mechanism contributing to this adaptive resistance, they suggested that the most likely cause was the presence of active multidrug efflux pumps. Resistance to multiple antimicrobials has been linked to mutations in drug-efflux systems such as the AcrAB multidrug efflux pump observed in *E. coli* [58], [59], as well as to mutations in the transcription factors controlling these systems, such as SoxS [60], MarA [61] and ROB [62]. These findings support the important role that sub-MICs of antimicrobial drugs have on the selection of multidrug resistant bacteria. Additionally, the small percentage of pansusceptible isolates in DR calves (6%) compared to NR calves (46%) is a reflection of the effect that sub-MICs of antimicrobials has on the promotion of resistance genes between commensal *E. coli*.

Independent of feeding treatment group, calves in the study had a peak in the proportion of resistant *E. coli* at one to two weeks of age for the majority of the antimicrobial drugs tested (Fig. 2). Similar findings were observed in a study by Berge et al. (2005), where calves 2 weeks of age were more likely to have multidrug resistant *E. coli* compared to calves 4 and 6 weeks old (OR = 29.8 and OR = 16.4, respectively). The introduction of resistant bacteria into the enteric microbiota depends on their ability to effectively compete with the indigenous microbiota [63]. Young calves lack a developed and diverse intestinal microflora, which could reduce the degree of protection against colonization by bacteria with a higher fitness cost, such as antimicrobial-resistant and pathogenic enteric bacteria [64]. Exposure of this tenuous enteric microbiota to sub-MICs of antimicrobials could increase the vulnerability of this microbiota to colonization by resistant bacteria. This was observed in our study, where from weeks one to six, calves in the DR group had a significantly higher proportion of isolates resistant to ampicillin, cefoxitin, and tetracycline compared to NR calves. However, because our study did not used quantitative methods, we could not determine whether this resulted primarily from decreased numbers of susceptible *E. coli*, an increase in the absolute number of resistant strains, or a combination of the two. Feeding calves milk with added sub-MICs of drugs since birth could have resulted in stress or perturbation beyond what the calves gastro intestinal microbiota could tolerate before its trajectory was changed towards a different equilibrium state [65]. This may result in a longitudinal change in the microbiota composition with an increase in the proportion of microbes carrying antimicrobial resistance genes that allow them to survive and reproduce under this continuous antimicrobial drug challenge. To test this hypothesis high throughput metagenomic methods would be needed. However, results from commensal *E. coli* suggest this as observed by a higher proportion of *E. coli* resistant to 3 or more antimicrobial drugs in the DR calves when compared to NR calves (Fig. 5).

Although at different rates, for most antimicrobial drugs in both milk feeding treatment groups the proportion of resistant *E. coli* decreased over the weeks after week 2. Calves are known to have a higher proportion of resistant *E. coli* when compared to older animals such as heifers and cows, which have an mature enteric microbiota [66], [67]. Although there were a greater proportion of resistant *E. coli* until the last week sampled in DR calves compared to NR calves, it is uncertain if as calves mature, animals from DR will continue to be hosts for a greater proportion of resistant *E. coli* when compared to animals from NR. Our study did not monitor animals beyond weaning and because of that we are unable to answer this question. However, studies in humans have revealed that treatment with antimicrobials can result in permanent changes to the microbiota that may result in the persistence of antimicrobial resistant bacteria [53], [68], [69]. Additional studies are needed to evaluate the impacts of feeding waste milk on the composition of the developing enteric microbiota of preweaned calves as they mature.

Based on the findings of our study, feeding waste milk to preweaned dairy calves is a practice that has the potential to at least temporarily increase the selection of resistance to drug of critical importance to both animal and human health. To eliminate the risk originating from feeding calves waste milk, an initial option would be to discontinue this practice at dairy farms. Some disadvantages include the possibility of polluting the environment with drug residues by disposing of the waste milk, disposal of a nutritious food source, and increase in the costs of feeding preweaned dairy calves. A reasonable assessment to determine whether to continue or discontinue feeding waste milk to dairy calves must take into account the health hazards to animals and humans, and economic advantages and disadvantages. An alternative to reduce the negative impacts of drug residues present in waste milk fed to calves is to use methods to degrade these drugs to concentrations below the minimum selective concentration (MSC) which may decrease the selection for a given resistance determinant. Some potential methods that could be used to reduce the concentration of drug residues in waste milk include heat treatment, time of storage, ion catalysis, and electrochemical methods. Heat treatment (120°C for 20 min) of milk containing beta-lactam drugs has shown to degrade 47% of amoxicillin, 84% of ampicillin, 53% of cloxacillin and 61% of penicillin G [70]. Biodegradation of ceftiofur has been shown to increase with the increase of temperature, with optimal degradation temperatures between 35°C and 45°C [71]. A study by Riediker et al. (2004) showed that penicillin G, amoxicillin, and ampicillin spiked in milk at a concentration of 0.01 ppm and stored for 6 days at 4°C suffered degradation in most cases of more than 50% of initial concentration [72]. Raw milk spiked with ceftiofur at a concentration of 0.01 µg/mL and stored for 14 days at 4°C retained 90 to 100% of the initial concentration, indicating that storage may not be the best option for reducing concentrations of cephalosporins in the milk [73]. Degradation of beta-lactams has also been shown in the presence of various metal ions where the ions catalyze the inactivation of the hydrolytic opening of β-lactams [74]–[76]. Electrochemical oxidation of raw milk with an initial concentration of oxytetracycline of 100 mg/ml has resulted in an 83% reduction of this drug after a 6h treatment [77]. Pasteurization of waste milk used to feed preweaned calves has been a recommended strategy to reduce bacterial contamination and limit the spread of disease, and furthermore may result in reduced concentration of certain antibiotics [78]. However additional studies must be conducted to measure the effectiveness of methods to reduce the concentration of drugs in the milk and take into account factors such as initial concentration of drug residues usually present in waste milk, final concentration of drugs after method application, and feasibility and costs of various methods.

In conclusion, feeding calves milk with residual concentrations (sub-MICs) of antimicrobial drugs from birth to weaning resulted in a higher proportion of *E. coli* isolates resistant to multiple antimicrobial drugs. Treatment of calves with sub-MICs of ampicillin, penicillin, ceftiofur, and tetracycline resulted in a greater proportion of *E. coli* resistant to ampicillin, cefoxitin, ceftiofur, streptomycin, and tetracycline. Additionally, isolates from DR calves had a significant decrease in susceptibility to ceftriaxone and ceftiofur when compared to isolates from NR calves. A significantly greater proportion of *E. coli* in DR calves was multidrug resistant when compared to isolates in NR calves. These findings stress that the exposure of *E. coli* to sub-MICs of antimicrobials in the milk has an important effect on the frequency of antimicrobial resistance in preweaned dairy calves.
